# The role of MLC901 in reducing VEGF as a vascular permeability marker in rats with spinal cord injury

**DOI:** 10.1016/j.amsu.2022.103344

**Published:** 2022-02-05

**Authors:** Wahyudi, Andi Asadul Islam, Mochammad Hatta, Willy Adhimarta, Muhammad Faris, Nasrullah Mustamir, Agussalim Bukhari, Prihantono, Cahyono Kaelan, Joko Hendarto, Naufal Hilmy Imran, Rohadi Muhammad Rosyidi

**Affiliations:** a Faculty of Medicine and Health Sciences, Muhammadiyah Makassar University, Makassar, Indonesia; b Doctorate Program, Faculty of Medicine, Hasanuddin University, Makassar, Indonesia; c Departement of Neurosurgery, Faculty of Medicine, Hasanuddin University, Makassar, Indonesia; d Molecular Biology and Immunology Laboratory, Faculty of Medicine, Hasanuddin University, Makassar, Indonesia; e Departement of Neurosurgery, Faculty of Medicine, Airlangga University, Surabaya, Indonesia; f Departement of Nutritional Sciences, Faculty of Medicine, Hasanuddin University, Makassar, Indonesia; g Departement of Surgery, Faculty of Medicine, Hasanuddin University, Makassar, Indonesia; h Departement of Neurology, Faculty of Medicine, Hasanuddin University, Makassar, Indonesia; i Departement of Community and Prevention Medicine, Faculty of Medicine, Hasanuddin University, Makassar, Indonesia; j Departement of Neurosurgery, Medical Faculty of Mataram University, West Nusa Tenggara General Hospital, Mataram, Indonesia

**Keywords:** *Spinal cord injury*, *MLC 901*, *Regulation of VEGF level*, *Increased vascular permeability*, *Tissue edema*

## Abstract

**Background:**

Damaged neural tissue caused by SCI could induce vascular endothelial growth factor (VEGF) that can worsen the condition in the late phase by increasing vascular permeability, thus inducing tissue oedema, which can worsen the infarction. MLC 901 has been widely used in Asia for stroke patients because its mechanism is known to down-regulate VEGF levels in ischemic tissue.

**Methods:**

Ten Sprague-Dawley rats were used in this experiment. To create a severe spinal cord injury in animal models. The animals were then randomly divided into two groups. MLC 901 was given to the first group, which was the intervention group, and placebo to the second group, which was the control group.

**Results:**

This study showed a decrease in the mean VEGF mRNA expression in the group given MLC 901 compared to the control group, which had a very high mean VEGF mRNA expression starting after 1 h of administration of MLC 901 until day 14 after spinal cord injury. In addition, there was a decrease in VEGF levels in the MLC 901 group compared to the control group from 3 h after spinal cord injury (1 h after MLC 901 administration) to 14 days after spinal cord injury.

**Conclusion:**

It can be concluded that administration of MLC 901 can reduce vascular permeability, one of the mechanisms that is thought to occur is to reduce VEGF levels. MLC 901 also maintains the neuroprotective effect provided by VEGF by maintaining this level above the basal level until day 14.

## Highlights

• Experimental study on mice subjects to with spinal cord injury.
• This Research to Evaluate of VEGF level in rats with spinal cord injury and also in VEGF gene mRNA expression between treatment (MLC901) group and control group in rats with spinal cord injury.
• MLC 901 also maintains the neuroprotective effect provided by VEGF by maintaining this level above the basal level until day 14.

## 1 Introduction

Spinal Cord Injury (SCI) is a neurological disease that causes motor, sensory, and autonomic dysfunction. It has high mortality and morbidity worldwide, and there are no currently effective therapeutic interventions which cause not only permanent neurological deficits but also large social and economic burdens for millions of individuals and families worldwide [[1], [2], [3]]. It was estimated that the incidence rate of SCI was between 52 and 54 cases per 1,000,000 between 1993 and 2012. Men had a higher incidence of SCI: 78% of new SCI cases were happened among men. Interestingly, the median age of SCI has become older (43 years in 2019 vs 29 years in the 1970s), this is due to the increasing proportion of SCI caused by falls among the growing elderly population. The main causes of SCI were traffic accidents (59.5%) and falls (37.8%). Most countries reported SCI causing more paraplegia than quadriplegia, with the exceptions of China, Japan, and Thailand, where the situation was reversed [4,5].

SCI is divided into primary and secondary injury phases, wherein the primary injury phase originates directly from physical trauma to the spinal cord from various mechanisms including compression, bruising, transection, and shear forces. Mechanical injury leads to a cascade of biological events, described as ''secondary injury''. Due to changes in the acute injury environment in the first 48 h after SCI, these secondary spinal cord injury processes include apoptosis, ischemia, ion-mediated cell damage, excitotoxicity, neuroinflammation, mitochondrial dysfunction, and oxidative cell damage [[6], [7], [8], [9]].

Vascular damage and breakdown of the blood spinal cord barrier (BSCB) are universal consequences of SCI both clinically and in animal models. After mechanical disruption of the capillaries at the time of injury, blood-borne molecules and cells easily cross into the injured parenchyma. In addition, damage to BSCB will increase vascular permeability, which will cause macrophages to infiltrate from injured blood vessels and accumulate in the microenvironment of spinal cord tissue, these macrophages will then express more cytokines and chemokines that further increase the permeability of BSCB. Furthermore, the accumulation of water in cells and extracellular compartments exacerbates spinal cord tissue edema [[10], [11], [12]].

Under hypoxic conditions, neural tissue is able to induce protective mechanisms to limit cell damage and thereby increase survival. One of these factors is VEGF which is upregulated after hypoxia [13,14]. VEGF was originally described as a vascular permeability factor because of its ability to induce vascular leakage. VEGF has mitogenic and anti-apoptotic effects on endothelial cells, increases vascular permeability, and promotes cell migration. In addition, VEGF also plays a role in tumor formation [[15], [16], [17], [18], [19], [20]]. A study by Benton and Whitemore found a profound exacerbation of spinal cord lesion volume in a VEGF-injected animal model of SCI rats [21]. In addition, VEGF also has a role in increasing the permeability of microvessels after brain hypoxia and functions as a potent mediator of vascular permeability [20,22,23]. VEGF and its receptors are also expressed in several non-neoplastic pathological processes, including hyperpermeability to circulating plasma proteins, retinopathy, rheumatoid arthritis, psoriasis, and other inflammatory conditions. In addition, inhibition of VEGF has a role in the treatment of neovascular ocular disease, and may be beneficial in other neurologic disorders involving impaired blood brain barrier (BBB) or excessive angiogenesis [20,24].

MLC 901 (Neuroaid II) is a combination of 9 herbal components and is indicated as a treatment for stroke recovery which is widely used in China and many countries in Asia. MLC 901 has neuroprotective and neuroproliferative effects and has been extensively described during in vitro and in vivo experiments using animal models. The remarkable effect of MLC 901 lies in its neurogenesis and neurorestoration effects rather than its neuroprotective effects [[25], [26], [27]].

## 2 Materials and methods

This research was conducted using 10 male Sprague-Dawley rats, weighing 250–300 g. According to the WHO's Research Guideline for Evaluating the Safety and Efficacy of Herbal Medicines, the sample size should be a minimum of 5 individuals per group, with a 10% drop-out reserve (1 head).The ethical standard for animal research was also considered before deciding the sample size. The Sprague-Dawley rats were adapted for 1 week before being treated. During adaptation, male Sprague-Dawley rats received basic feed and standard maintenance. Five male Sprague-Dawley rats were used as the treatment group (T). Five male Sprague-Dawley rats were used as the control group (C). MLC 901 was diluted in saline (as vehicle) at a concentration of 75 mg/ml (stock solution) and incubated under agitation for 1 h at 37 °C. The solution was then filtered with a 0.22-m filter. For the time window of protection, rats were intraperitoneally injected with a single dose of MLC 901 at a concentration of 0.075 mg/ml (in a bolus of 500 ml) 2 h after SCI, followed by an oral administration in drinking water (10 mg/ml) until the sacrifice of the animals.

### 2.1 Study design

True experimental laboratory research with a completely randomized research design was used in this study. On samples and treatments, controlled and measurable conditions were used. The research group was supposed to come from a population of experimental animals, and the study used a pre-test post-test control group design.

### 2.2 Animals

Experimental unit is serum and neuron cells in rat spinal cord Sprague-Dawley male at the level of the T2 vertebra. Physically healthy, white in color, 250 g in weight, 3 months old. Samples were obtained from the Laboratory of Animal Microbiology, Faculty of Medicine, Hasanuddin University and placed in a cage with controlled temperature and humidity (25 ± 1 °C.) with light and dark cycles every 12 h in the experimental animal unit, Laboratory of Microbiology and Molecular Biology, Faculty of Medicine, Hasanuddin University. All animal procedures have been approved by our local Ethics Committee (Number: 599/UN4.6.4.5.31/PP36/2020).

### 2.3 Surgical procedure

The surgical procedure was performed using aseptic techniques. Ten Sprague Dawley rats with spinal cord injury were randomly divided into two groups: (1) with MLC 901 administration, and (2) without MLC 901 administration (placebo). Anesthetics are performed with diluted ketamine (dose 3–10 mg/kgBW). The action taken is in the form of trauma given to the spinal cord at the T2 level by clamping using an aneurysm clip with modified clamping strength. Severe spinal cord injury will occur after clamping with a force of 35 g for 1 min [30].

Laminectomy was performed at T2 to expose the spinal cord. A dissecting hook, with similar curvature and thickness as the clip, was used to dissect the extradural plane between the dura and the adjacent vertebrae. With the clip held in the applicator in its opened position, the lower blade of the clip was passed extradurally anterior to the cord with avoidance of damage to the adjacent nerve roots. The clip was then rapidly released from the applicator to produce acute impact compression injury. The clip was left compressing the spinal cord for 1 min before removal with the applicator. We sutured the incision wound with non-absorbable 5.0 suture (silk) and treated it with topical antibiotics. All surgical procedures were performed aseptically by adhering to the principle of sterility. After the surgery and trauma, all animals were kept at room temperature for recovery and returned to their cages according to their groups [20,27,28,30].

### 2.4 Research procedure

1. Prior to SCI, blood was drawn from rats to measure VEGF gene mRNA expression and serum VEGF levels of experimental animals for all groups.
2. The rats were operated under general anesthesia (ketamine HCl 60 mg/kgBW and Xylazine 10 mg/kBW) intraperitoneally (IP).
3. Ampicillin (IM) antibiotic injection was given to hair and aseptic procedure with betadine in the back region.
4. A laminectomy was performed on the T2 vertebra and the dura mater was cleaned of the surrounding tissue using a dissector.
5. Compression is performed for 1 min using an aneurysm clip that has been modified so that it has a strength of 35 g to produce severe spinal cord injury.
6. The aneurysm clip is then released and the wound is closed again by means of stitches.
7. Blood samples were taken for VEGF gene mRNA expression and serum VEGF levels 2 h after SCI treatment (shortly before being given MLC901 intraperitoneal injection).
8. One hour after injection of MLC901 intraperitoneally, blood samples were taken from rats to measure VEGF gene mRNA expression and serum VEGF levels.
9. Subsequently, MLC901 was administered individually with a conversion dose of 68.4 mg/day until the termination of the experimental animals on day 14th.
10. On the 7th day after SCI treatment, the blood of the rats was taken back to measure VEGF gene mRNA expression and serum VEGF levels.
11. On the 14th day after the SCI treatment (before termination), the blood of the rats was taken back to measure VEGF gene mRNA expression and serum VEGF levels.

### 2.5 VEGF sample examination

Examination of VEGF samples was carried out by taking blood samples 5 times: before treatment, 2 h after treatment, 3 h after treatment (1 h after giving MLC 901 intraperitoneally), 7th day and 14th day after treatment. All blood samples were examined by VEGF Sandwich ELISA with Catalog No. Ls-F978 from Jansen Chimica, Beerse, Belgium, 10.846.79. Examination of VEGF gene mRNA expression using realtime PCR technique was carried out using a Realtime PCR machine (CFX Connect system, Biorad Laboratories, Real Time PCR 96 well 0.1 ml, USA).

## 3 Results

### 3.1 Subject characteristics

This study aimed to examine the benefits of MLC 901 in SCI cases and its relationship to VEGF ELISA and VEGF mRNA levels using a spinal cord injury model in rats (*Sprague Dawley*). Characteristics of the subjects, such as body weight, are listed in Table 1.

**Table 1:** Sprague Dawley rat body weight data.

	Treatment	Rat body weight (gram)	p-value
Mean	Control	264,40+/-8,35	0,48
	MLC 901	261,20+/-4,76	

### 3.2 VEGF ELISA levels

**Fig. 1:**
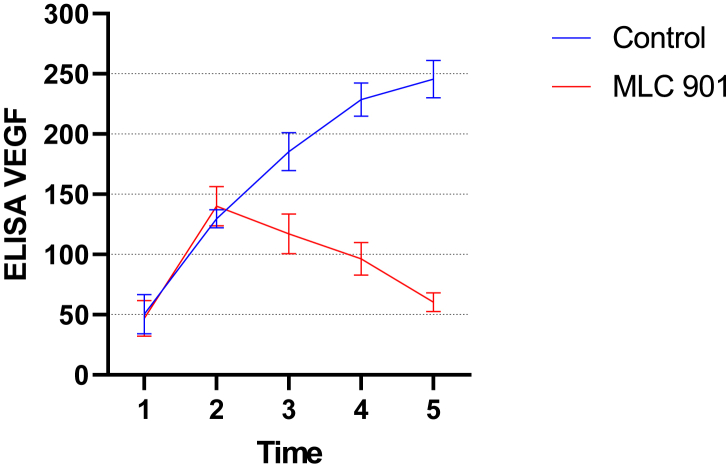
VEGF levels in experimental rats with and without MLC 901 treatment based on treatment time.

On days 7 and 14, the group given MLC 901 had lower VEGF levels than the placebo group (see Table 1). There was a significant difference in the levels of VEGF elisa in the MLC 901 group and the control group. On day 14 the group given MLC 901 had lower VEGF levels than on day 7. This was inversely proportional to the placebo group which had higher VEGF levels on day 14 than on day 7 (Table 2 and Fig. 1)**.**

**Table 2:** Effects of MLC 901 on VEGF levels in rats with spinal cord injury.

Time	Administration of MLC901	VEGF ELISA		Mean Difference	P
		Mean	Std. Deviation		
Before treatment	(+)	46,97	14,91	−3,65	0,74
	(-)	50,34	16,24		
2 h after treatment	(+)	140,12	16,32	10.53	0,23
	(-)	129,59	7,46		
3 h after treatment (1 h after administration of MLC901)	(+)	117,04	16,53	−68,41	0,00
	(-)	185.45	15,70		
7 days after treatment	(+)	98,21	11,38	−130.5	0,00
	(-)	228,71	13,94		
14 days after treatment	(+)	60,40	7,87	−91,56	0,00
	(-)	152,96	15,64		

Statistical test with independent T-test, with significance p < 0.05.

### 3.3 mRNA gene VEGF

**Fig. 2:**
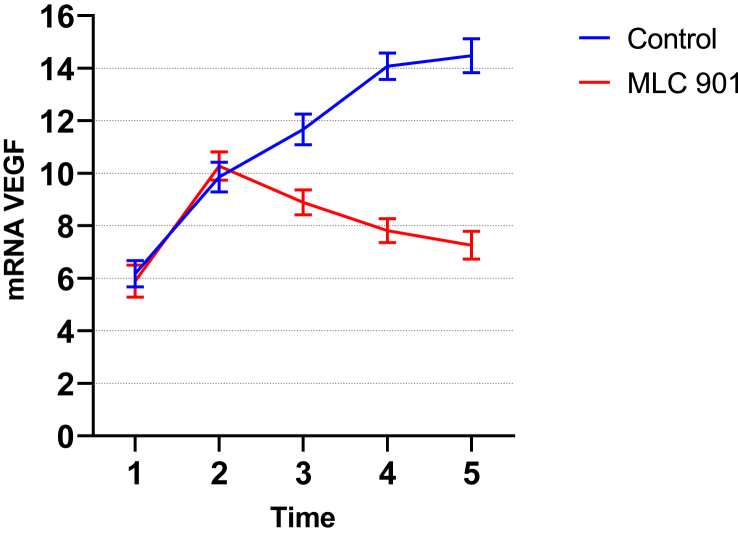
VEGF mRNA expression in rat experimental with and without MLC 901 treatment based on treatment time.

Directly proportional to the results of VEGF ELISA levels, the results of the RT PCR VEGF GENE examination in the placebo group were much higher in the control group on days 7th and 14th compared to the MLC 901 group. The same thing also happened on the results of the examination on day 14th which were higher from day 7 in the placebo group, while in the MLC 901 group VEGF mRNA on day 14th decreased compared to day 7th (Table 3 and Fig. 2).

**Table 3:** Effects of MLC 901 on VEGF gene mRNA expression in rats with spinal cord injury.

Time	Administration of MLC901	mRNA gen VEGF		Mean Difference	P
		Mean	Std. Deviation		
Before treatment	(+)	5,89	0,62	−0,28	0,45
	(-)	6,17	0,50		
2 h after treatment	(+)	10,28	0,54	0,42	0,26
	(-)	9,86	0,56		
3 h after treatment (1 h after administration of MLC901)	(+)	8,89	0,47	−2,78	0,00
	(-)	11,67	0,58		
7 days after treatment	(+)	7,81	0,45	−6,26	0,00
	(-)	14,07	0,50		
14 days after treatment	(+)	7,26	0,53	−7,21	0,00
	(-)	14,47	0,65		

Statistical test with independent T-test, with significance p < 0.05.

### 3.4 Statistical Analysis

The data were processed and analyzed using Excel 2013 and SPSS version 22. The effect of administered MLC 901 which are VEGF mRNA expression and VEGF levels will be displayed in the form of mean (standard deviation) with confidence interval (95% CI). To see the difference in mean VEGF mRNA expression and VEGF levels in the treatment group and the control group based on different observation times, ANOVA Repeated Measurement test was performed. To see a significant difference in the mean between groups from the Repeated Measurement ANOVA test, a post hoc Least Significantly Difference (LSD) test was carried out.

## 4 Discussion

Research that has been conducted on the role of MLC 901 in ischemic stroke cases in rats showed that MLC 901 increased survival rate and decreased cerebral infarct area after ischemia and reperfusion. The study showed that the levels of VEGF detected in the ELISA test increased in the vehicle group that experienced middle cerebral artery occlusion (MCAO). The increase in VEGF occurred at 24 h and 3 days after ischemia and tended to return to basal levels on day 14. This was inversely proportional to the group given MLC 901, where in the first 24 h after MCAO VEGF levels were seen to decrease and increase on day 4. 3 and 14. From these studies it was found that MLC 901 promotes angiogenesis in response to MCAO by modulating the expression of angiogenesis-related factors such as Hif1α, EPO, VEGF and Ang1/Ang2. These factors are known to not only mediate endothelial cell proliferation, but also regulate vascular differentiation, regression and permeability [31]. This study is in line with research conducted by Quintard et al., the study explained that administration of MLC 901 in traumatic brain injury significantly stimulated VEGF expression on the seventh day after injury, both in the hippocampus and cortex of experimental animals compared to the vehicle group [29].

Although VEGF is an important signaling molecule closely related to angiogenesis, axonal guidance, neuroprotection, survival and migration of Schwann cells and proliferation of astrocytes, microglia and neural stem cells [32]. On the other hand, administration of VEGF can increase the permeability of BSCB in the acute and sub acute period even to the chronic phase [33]. High VEGF levels, as a result of upregulation by severe ischemia or from systemic VEGF administration, can exacerbate tissue damage through increased BBB leakage resulting in poststroke brain edema and life-threatening intracranial hypertension. In addition, damage to the BBB by VEGF can increase glutamate and albumin extravasation, which activates astrocytes and disrupts K+ homeostasis in brain parenchyma, leading to neuronal hyperactivity and stress. Consistent with these observations, administration of VEGF immediately after stroke increased vascular leakage and cerebral infarction, whereas blockade of VEGF earlier after stroke reduced brain edema and infarct size. Other studies have also indicated an association of cerebral microbleeds with increased VEGF levels in patients with cerebral large artery disease [24,34]. Meanwhile, in a study using a young adult mouse model of diabetes, it has been shown conclusively that inhibiting the VEGF 2 receptor (VEGFR2) improves functional outcomes and impaired BBB after stroke [35].

As mentioned above, VEGF has both positive and negative hypotheses regarding its effect on neurologic dysfunction, particularly SCI. However, this study supports the hypothesis that high VEGF levels may have a negative effect on SCI conditions, which we assume is a role for high VEGF levels on vascular permeability after SCI. Interestingly, the results of our study also showed that in an animal model given MLC 901 VEGF levels starting from 2 h after treatment until day 14 remained above the basal level but did not experience a spike as high as VEGF levels in the placebo group, so we assumed that in the group given MLC 901 retains the neuroprotective effect of VEGF against SCI in this animal model. The limitation of this study is that the research was carried out on experimental animals so that it needs to be continued in the future with clinical research involving neuroprotective effect of VEGF against SCI and biomarkers.

## 5 Conclusion

From the results described above, it can be concluded that administration of MLC 901 can reduce vascular permeability by reducing VEGF levels. This can be seen in the SCI group given MLC 901 which had lower VEGF levels than the placebo group. However, MLC 901 also maintains the neuroprotective effect provided by VEGF by maintaining this level above the basal level until day 14. There are some limitations to this study. Thus, further research is needed to fully elucidate the neuroprotective impact of MLC 901 on SCI and its association with VEGF.

## Ethical approval

All procedures on experimental animals have been approved by the ethics committee by the Ethics Commission Faculty of Medicine, Hasanuddin University, (Number: 599/UN4.6.4.5.31/PP36/2020)

## Sources of funding

No funding or sponsorship.

## Author contribution

WYD, NHI, AAI, MH and WA wrote the manuscript and participated in the study designed. FAZ, NAL, PRI, AGB and CAH participated in the study designed. WYD, AAI, MH, WA and RHA drafted and revised the manuscript. WYD, NHI, MH and FAZ performed spinal cord injury treatment and surgery. WYD, JH and NHI performed bioinformatics analyses and revised the manuscript. All authors read and approved the final manuscript.

## Registration of research studies

1. Name of the registry:
2. Unique Identifying number or registration ID:
3. Hyperlink to your specific registration (must be publicly accessible and will be checked):

None.

## Guarantor

Wahyudi.

## Consent

This manuscript does not involve human participants, human data, or human tissue.

## Declaration of competing interest

The authors declare that they have no conflict of interests.
